# Precision Medicine in Neurodegenerative Diseases: Some Promising Tips Coming from the microRNAs’ World

**DOI:** 10.3390/cells9010075

**Published:** 2019-12-27

**Authors:** Nicoletta Nuzziello, Loredana Ciaccia, Maria Liguori

**Affiliations:** 1 National Research Council, Institute of Biomedical Technologies, Bari Unit, 70126 Bari, Italy; 2 Department of Biomedical Science and Human Oncology, University of Bari “Aldo Moro”, 70124 Bari, Italy

**Keywords:** microRNA, neurodegenerative diseases, precision medicine, pharmacoepigenomic, drug response, epidrug

## Abstract

Novel insights in the development of a precision medicine approach for treating the neurodegenerative diseases (NDDs) are provided by emerging advances in the field of pharmacoepigenomics. In this context, microRNAs (miRNAs) have been extensively studied because of their implication in several disorders related to the central nervous system, as well as for their potential role as biomarkers of diagnosis, prognosis, and response to treatment. Recent studies in the field of neurodegeneration reported evidence that drug response and efficacy can be modulated by miRNA-mediated mechanisms. In fact, miRNAs seem to regulate the expression of pharmacology target genes, while approved (conventional and non-conventional) therapies can restore altered miRNAs observed in NDDs. The knowledge of miRNA pharmacoepigenomics may offers new clues to develop more effective treatments by providing novel insights into interindividual variability in drug disposition and response. Recently, the therapeutic potential of miRNAs is gaining increasing attention, and miRNA-based drugs (for cancer) have been under observation in clinical trials. However, the effective use of miRNAs as therapeutic target still needs to be investigated. Here, we report a brief review of representative studies in which miRNAs related to therapeutic effects have been investigated in NDDs, providing exciting potential prospects of miRNAs in pharmacoepigenomics and translational medicine.

## 1. Introduction

The great advances in high-throughput next generation sequencing (HT-NGS) technologies and large-scale computation facilities have enabled a deeper investigation also in neurodegenerative diseases (NDDs), providing a more comprehensive overview of these complex and multifactorial disorders and promoting the development of a precision medicine approach, e.g., for their monitoring and cure [1]. NDDs are mostly characterized by progressive neuronal loss in the central nervous system (CNS) causing a decline in several CNS functions [2]. The most known NDDs include Alzheimer’s disease (AD), Parkinson’s disease (PD), Huntington’s disease (HD), and amyotrophic lateral sclerosis (ALS), in which neurodegeneration represents the main pathological hallmark. However, in the last decades other multifactorial disorders have been added in the NDDs category, due to evidences of consistent neurodegenerative components together with other pathogenic processes, like the autoimmune neuroinflammation in multiple sclerosis (MS) [3].

Among the new players identified, microRNAs (miRNAs) are certainly the most extensively studied because of their potential role as biomarkers for diagnosis, prognosis, and response to treatment. Indeed, the discovery of miRNAs and their implication in several CNS-related disorders have opened up new prospects in the search for biomarkers and therapeutic targets, in order to monitor the disease progression and to possible guide individualized treatments [1,4,5,6,7].

MiRNAs are a class of small non-coding RNAs that are involved in the regulation of gene expression at the posttranscriptional level by base-pairing and repress translation of target mRNAs [8]. The official miRNA repository database (miRbase; Release 22.1) currently lists 1917 precursor miRNAs and 2654 mature miRNAs in the human genome that act in concert to regulate up to approximately 60% of all coding genes [9]. Specifically, miRNAs act as sequence-targeting guides that associate with the RNA-induced silencing complex (RISC) to knockdown the mRNAs. In addition, they can also modulate biological processes by targeting competing endogenous RNAs (ceRNAs) which carry the same miRNA response elements (MREs) [10] and by generating negative or positive feedback loops with transcription factors [11].

Emerging evidences demonstrated that dysfunctional miRNA regulatory networks have been associated to several NDDs [12,13,14,15,16]. In the human brain, miRNAs act not only as fine-tuners but also as master regulators of neuronal circuit development, maturation, and function, and it is able to influence processes such as cell-fate determination, cell migration, neuronal polarization, cognition, synapse formation, and plasticity [17]. Of note, the discovery of miRNAs in body fluids (blood, saliva, urine, serum, and cerebrospinal fluid, CSF) proved to be particularly relevant in NDDs, in which the analysis of peripheral biomarkers could be helpful e.g., for a more accurate detection of a given disease onset [18]. More importantly, recent studies in the field of neurodegeneration showed unexpected miRNA-mediated mechanisms in the regulation of drug response and efficacy: (i) miRNAs can regulate the expression of pharmacology-related target genes and/or (ii) pharmacological therapies can restore altered miRNA expression levels [18,19]. Indeed the involvement of miRNAs in response to drugs is part of an emerging branch of pharmacogenomics, which is referred to as *pharmacoepigenomics* [20]. The knowledge of miRNA pharmacoepigenomics not only may provide novel insights into the interindividual variability to drug disposition and response, but also offers new clues to develop more effective treatments [21]. 

In this review, we first reported some representative studies in which miRNAs possibly involved into therapeutic effects have been investigated in some NDDs. In particular, we detailed the existing data on AD, PD, and MS, as they are the only NDDs with consistent literature regarding the issue of interest. To our knowledge, there are no studies on miRNomic profiles in response to treatments for other NDDs like HD and ALS. Finally, we focused on exciting potential prospects of miRNAs in pharmacoepigenomics and translational medicine. 

## 2. MiRNAs as Pharmacoepigenomic Targets for NDDs

The rapid and major advances in epigenomics are impacting the modern pharmacology, giving rise to a burgeoning field known as pharmacoepigenomics, that is a genome-wide scale study of the epigenetic basis of individual variations of the drug response [22]. Epigenomics refers to genome-wide studies on three interacting molecular mechanisms: DNA methylation, modification of histones in chromatin, and RNA-mediated regulation of gene expression via non-coding RNAs, such as miRNAs, circular RNAs, and long non-coding RNAs [23]. Over the last two decades, epigenomics has begun to exert a great impact in different areas such as the study of CNS development, learned behavior, neurotoxicology, cognition, addiction, and lately of many neurological and neurodegenerative pathologies [24]. DNA methylation, i.e., the addition of a methyl group on the fifth carbon at cytosine, is the predominant epigenetic modification of eukaryote genomic DNA. It occurs in cytosine–phosphate–guanine (CpG) islands and in non-CpG (CpH) sites [25]. CpH methylation is present predominantly in the neuronal genome and accumulates during synaptogenesis [26]. Among the others, Lister et al. [27] reported a whole-genome base-resolution analysis of DNA cytosine modifications and transcriptome analysis in the frontal cortex of human and mouse brains at multiple developmental stages. Their results highlighted the role of the epigenome in pathological disruptions of the neural circuits [27]. Additionally, the imbalance in histone acetylation levels and consequently the dysfunction in transcription have been associated with a wide variety of NDDs [28]. In vitro and in vivo animal models and post-mortem analysis of brains derived from NDDs patients reported overexpressed level of histone deacetylases (HDACs), thus encouraging new therapeutic approaches in this direction [29,30,31].

Finally, miRNAs mediated post-transcriptional regulation represents a newly recognized mechanism that attracted much interest in recent years. Two models of miRNA-mediated therapeutic effects have been proposed: direct and indirect. The first model reveals that most of the approved drugs for NDDs can directly restore the expression level of altered miRNAs and possibly contribute to their therapeutic effect [18]. The second model suggests that miRNAs may influence the drug efficacy by regulating the expression of genes involved in drug absorption, distribution, metabolism, and excretion (ADME) [32,33]. This epigenetic regulation of miRNAs in ADME genes could justify why different patients may respond differently to the same treatment. Understanding the factors that cause inter-individual differences in the efficiency of a given drug metabolism is mandatory for the possibility to develop the so-called *personalized* or *precision medicine*, as well as for the promotion of a more efficient drug development [33], even though this process may complicate the already complex molecular mechanisms of drug activity.

**Figure 1:**
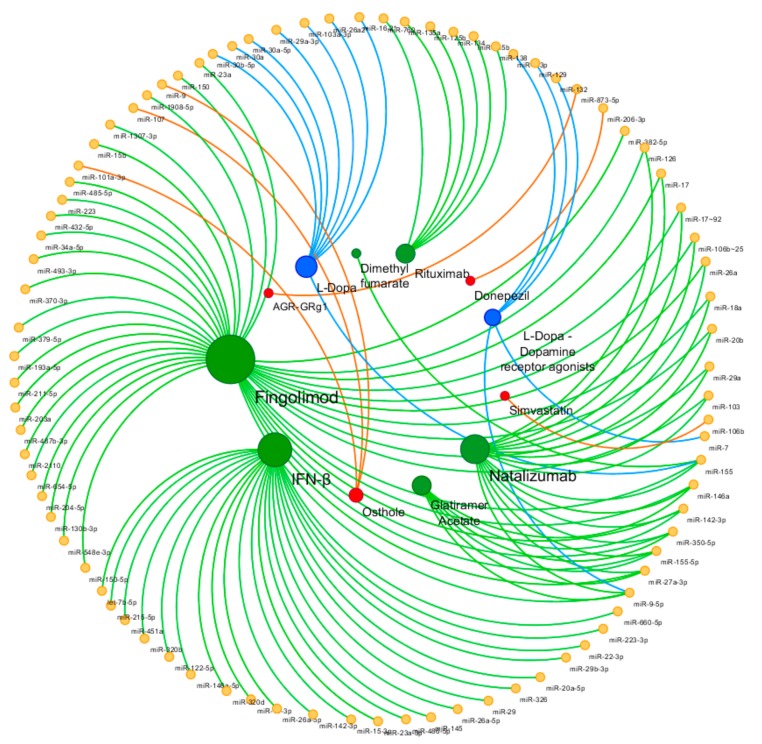
Circular view of microRNAs (miRNAs) and related drugs. Yellow nodes represent miRNAs, green nodes represent multiple sclerosis (MS) disease-modifying therapies, blue nodes represent Parkinson’s disease (PD) treatments, and red nodes represent AD treatments. The color of edges is associated to neurodegenerative diseases (NDDs), and the size of the nodes is proportional to the degree of the nodes (number of incoming and outcoming edges). The network is visualized using Cytoscape v3.7.1. (Institute for Systems Biology, Seattle, WA, USA).

In the following section, we attempt to provide a brief review of the so far investigated miRNomic profiles in response to the available conventional (and non-conventional) treatments of some NDDs (Table 1). The attention was addressed mainly to AD, PD, and MS, for which we were able to find consistent and detailed studies on the issue of interest. Figure 1 shows the miRNAs involved in drug response in the investigated NDDs.

## 3. Alzheimer’s Disease

Alzheimer’s Disease (AD) is the most common neurodegenerative disorder [34] and a progressive chronic condition representing the leading cause of dementia among people aged 65 and older [35]. AD is characterized by a progressive decline of cognitive abilities, with behavioral and psychotic symptoms that leads to premature loss of personal autonomy and death [36]. The major neuropathological hallmarks are extraneuronal senile plaques (mainly constituted of aggregated amyloid β-peptide) and intraneuronal neurofibrillary tangles (NFTs) (rich in abnormally phosphorylated tau) [35,36,37].

The AD genetic background discriminates between early-onset familial AD (FAD), other cases of sporadic early onset AD, and late-onset AD. The rare cases of early-onset FAD are caused by high-penetrant mutations in genes coding for amyloid precursor protein (APP), presenilin 1 (PSEN1), and presenilin 2 (PSEN2). Late-onset AD is multifactorial and associated with many different genetic risk loci (>20), with the apolipoprotein E ε4 allele being a major genetic risk factor [38].

Although very recent promising steps in the direction of finding effective treatments, to date there is no cure for AD; however, few drugs and care strategies seem to improve or stabilize some symptoms, leading to positive changes especially for the quality of life of patients and their families [39]. The pharmacological therapies currently available for AD patients include two classes of drugs [40]: (i) Donepezil, rivastigmine, and galantamine, which are cholinesterase inhibitors recommended for patients with mild, moderate, or severe AD dementia [40,41]; (ii) memantine, either a non-competitive N-methyl-D-aspartate (NMDA) receptor antagonist and a dopamine agonist approved for the treatment of patients with moderate-to-severe AD [40,41,42]. Among these drugs, possible effects of donepezil involving miRNAs have been observed by Wang et al. [43] in APP/PSEN1 transgenic mice. They found that the level of miR-206-3p expression was enhanced in the animal model of AD, while the treatment with donepezil decreased this level in the hippocampus and cortex of APP/PSEN1 transgenic mice [43]. Since increased miR-206 levels seem to contribute to the pathology of the disease by downregulating a neurotrophin called brain-derived neurotrophic factor (BDNF) [44], this finding suggests that miR-206 could be a pharmacological target for developing novel therapeutic approaches [43].

No effective therapies are currently available for treating the neurotoxicity observed during the disease [45]. Furthermore, most of the aforementioned drugs are only able to relieve some AD symptoms while they may have some deleterious side effects, like agitation/aggression, confusion, dizziness, falls, respiratory infections, and gastrointestinal symptoms [46], so the urgency to develop effective therapeutic agents for treating the disease is more than clear.

Finally, several reports demonstrated that Statins multiple effects, including anti-inflammatory properties, may be beneficial in AD; indeed, researches suggested that the inflammation does contribute to the pathogenesis of the disease [47]. Some clinical data provided evidence of a possible association between the use of Statins and the reduced risk of AD. In a study conducted in order to verify these evidences, Huang et al. [47] observed that Simvastatin ameliorated the memory deficits both in AD patients and in the animal model of AD. They also found that one specific Statin (Simvastatin) inhibited the apoptosis of neural stem cells and improved the survival of the neurons by modulating the expression of miR-106b [47].

Besides the traditional medicine, the pharmacological activities of some natural substances have been also investigated, as they also exerted significant impact in the expression levels of some miRNAs potentially implicated in the AD pathogenesis. Osthole (7-methoxy-8-isopentenoxycoumarin, C_15_H_16_O_3_), a natural Coumarin first derived from Cnidium plant, is an active constituent extracted from some medicinal plants, commonly used in the clinical practice of traditional Chinese medicine [48,49]. In literature there are evidences that Osthole has several pharmacological effects, such as neuroprotection, osteogenesis, anti-inflammation, anti-apoptosis, and anti-oxidation [49,50,51,52], and in fact it showed a neuroprotective potential in AD [53]. Although the exact molecular mechanism has not yet been fully elucidated [48], some studies suggest a possible involvement of miRNAs in its therapeutic efficacy. Li et al. [45] demonstrated a protective role of Osthole in the neuronal synapse, since it significantly increased the expression of miR-9 in APP-overexpressed cells [45]. Later on, they hypothesized a cross-talk between miR-9 and the Notch signaling pathway in AD models and observed that Osthole may promote the NSCs differentiation via the upregulation of miR-9 and the subsequent inhibition of the Notch signaling pathway in APP-expressing cells [54]. Jiao et al. [48] reported a neuroprotective activity of Osthole that involves the upregulation of miR-107 in AD. Furthermore, they observed that the administration of Osthole to APP/PS1 transgenic mice significantly improved the cognitive function by decreasing the Aβ contents in the hippocampal and cortex region of the brain [48]. MiRNA-101a-3p was also reported as Osthole-mediated miRNA in AD [52], since it seemed to improve the learning and memory ability of APP/PS1 mice, to increase the miRNA-101a-3p expression and to reduce the levels of APP at the same time [52].

Acori graminei Rhizoma (AGR) and Ginsenoside Rg1 (GRg1) are other traditional Chinese herbal drugs experimented in cognitive impairments; in particular, AGR has been used to treat senile dementia, while GRg1 showed neuroprotective role in AD [55]. In a study aimed to identify the therapeutic effect of GRg1 and AGR in the animal model of AD, Shi et al. [55] found that the combination of GRg1 and AGR promoted the expression of miR-873-5p, suggesting that these non-traditional drugs might have some benefit in the AD treatment with a potential mechanism in part mediated by miR-873-5p.

## 4. Parkinson’s Disease

Parkinson’s disease (PD) is the second-most common neurodegenerative disease [56] characterized by both motor and non-motor system symptoms. The disease occurs mostly in the older ages but an early-onset PD has been also described [57]. Resting tremor, rigidity, bradykinesia, and loss of postural reflexes are the classical parkinsonian motor symptoms [58]. They are most likely the result of the dopamine deficiency caused by the prominent death of dopaminergic neurons in the *pars compacta* of the *substantia nigra,* which is the typical pathological hallmark of the disease [59]. PD patients may also complain non-motor symptoms, including cognitive impairment, depression and sleep disorders, at any stage of the disease, even before the onset of the motor signs [58,60].

Although the full etiology of PD is still partially unknown, the development of the disease seems to rely on the combination of genetic and environmental factors [59,61], as in fact several causative genes seem to be implicated [62]. Currently no clinically validated biomarkers for PD monitoring has been identified [62]. However, there are evidences that miRNAs can be associated with PD pathophysiology as well, since they seem to be involved in the disease progression by regulating different PD-related genes [14,63,64,65].

Current therapies for PD are able to treat most of the symptoms quite effectively [59]. Since the main cause of PD is the dopamine deficiency, the pharmacological treatment of the disease is closely related to the restoration of this neurotransmitter’s levels [66].

l-dopamine (l-dopa), a precursor of dopamine, was the first symptomatic treatment for PD [67]. Although it is the most effective medication, in some cases other therapeutic approaches, e.g., with monoamine oxidase type B inhibitors, amantadine, anticholinergics, β-blockers or dopamine agonists, may be used as first-line therapy to avoid some motor complications related to the administration of l-dopa, such as dyskinesia, impulsive and compulsive behaviors, and hallucinations [68]. However, as far as we know, changes of the expression levels of selected miRNAs have been investigated mostly during the l-dopa treatment. Schwienbacher et al. [69] profiled the expression levels of five candidate miRNAs (miR-29a-3p, miR-29b-3p, miR-30a-5p, miR-30b-5p, and miR-103-3p) in three different data sets (l-dopa-treated PD patients, drug-naïve PD patients, and unaffected controls). They reported a trend of upregulation for miR-30b-5p in drug-naïve patients, while the expression of miR-30a-5p was upregulated in plasma samples of l-dopa-treated PD patients [69]. A significant overexpression of miR-29a-3p, miR-30b-5p, and miR-103a-3p in l-dopa-treated PD patients was also found by Serafin et al. [62].

Alieva et al. [70] investigated the expression levels of different miRNAs in PD patients treated with different medications, including dopamine receptor agonists, l-dopa, and amantadine. They observed that the levels of miR-7, miR-9-3p, miR-9-5p, miR-129, and miR-132 in treated PD patients were higher than those of the controls [70]. A similar result was obtained in another study [58], in which the l-dopa treatment induced significant changes in the expression profile of several miRNAs. In particular, they found that the expression levels of miR-16-2-3p, miR-26a-2-3p, and miR-30a were able to discriminate between treated and untreated patients [58]. In contrast to these studies reporting that l-dopa treatment could increase the expression levels of different miRNAs, Caggiu et al. [61] observed a significant decrease in miR-155 in patients treated with the higher doses of l-dopa.

Although further studies are needed to confirm these data, it seems that miRNAs are very sensitive at least to some of the treatments currently used in the clinical practice of PD, so also in this disease they may became the targets of innovative therapeutic strategies [70].

## 5. Multiple Sclerosis

MS is a heterogeneous neurological disorder of the CNS characterized by autoimmune inflammation coupled to demyelination and neurodegeneration [3]. MS most often follows a relapsing-remitting course (RRMS), in which acute episodes of clinical/MRI activity are followed by partial or complete recovery [71]. Over the time course (around 10−15 years from the onset), some of the RRMS patients (20%−30%) complain of a progressive accumulation of disability, possibly due to neurodegeneration: It means that they are converting into the so-called secondary progressive MS (SPMS). Finally, in a minority of patients (7%−10%) the clinical pattern is characterized by a chronic progressive deterioration of the neurological abilities from the very onset of the disease, which is referred to as primary progressive MS (PPMS) [72].

To date, there is no definitive treatment available for MS, and the current therapeutic strategy is mostly addressed to reduce the risk of relapses and potentially the subsequent disability progression [73,74,75,76]. Patients are treated with disease-modifying therapies (DMTs), which have different mechanisms of action that aim to suppress or modulate the dysregulated immune system, limit CNS inflammation, and prevent relapses and new CNS lesions [73]. Currently, a total of 18 DMTs are approved by the FDA, but most of them are prescribed for RRMS patients. Recently, a new treatment (Siponimod) has received the first positive approval from the European Medicine Agency (EMA) specifically for treating the SPMS patients, whereas Ocrelizumab is currently the only DMT that is approved for patients with PPMS.

**Figure 2:**
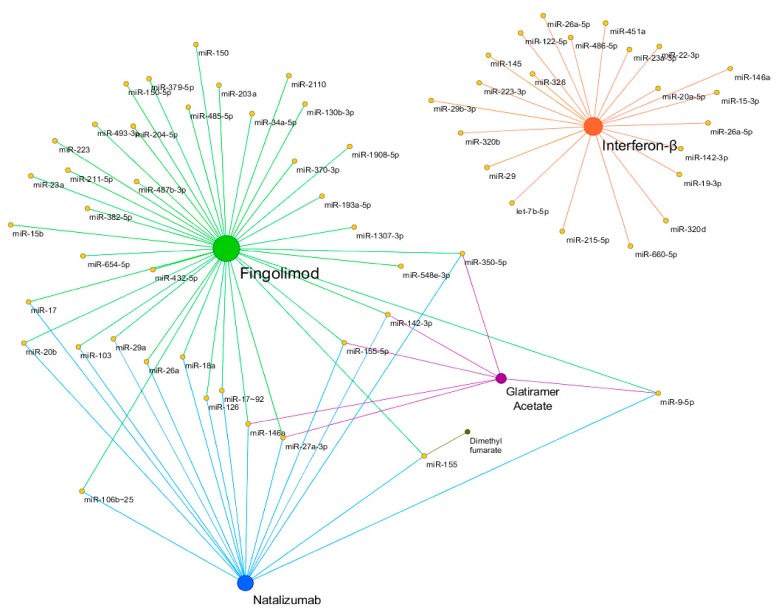
Schematic representation of miRNA pharmacoepigenomics reported in MS by Cytoscape v3.7.1. Yellow nodes represent miRNAs. Fingolimod, Natalizumab, Glatiramer Acetate, Interferon-β, and Dimethyl fumarate, are shown in green, blue, purple, orange, and brown, respectively. The size of the nodes is proportional to the degree of the nodes. It is worthy to mention that, among the more frequently used disease-modifying therapies (DMTs) in relapsing-remitting course (RRMS), Fingolimod, Glutiramer Acetate, and Natalizumab seem to significantly impact the expression levels of common miRNAs (and possibly target genes), while their mechanisms of action are quite different (see the text). Indeed, this observation may suffer from some biases due to e.g., the selection of the investigated miRNAs, the molecular methods used, etc., so more definitive conclusions can be drawn after planned studies.

Increasing evidence showed that several DMTs were able to restore the expression levels of miRNAs dysregulated in MS patients. Figure 2 shows a schematic representation of miRNA pharmacoepigenomics reported in MS. Interferon-β (IFN-β) and glatiramer acetate (GA) were the first two DMTs approved more than 20 years ago for the treatment of MS. IFN-β represents an efficacious first-line therapy whose mechanism of action lies in its ability to modulate the immune system activity, mainly by reducing the migration of peripheral T lymphocytes to the CNS [77]. The first longitudinal study on the miRNA expression changes in response to IFN-β showed that an up-regulation of IFN-β-responsive genes was coupled to a down-regulation of several miRNAs (including members of the miR-29 family) in peripheral blood mononuclear cells (PBMCs) of RRMS patients [78]. The involvement of miRNAs in response to IFN-β was pointed out by a clinical trial, where the participants received standard IFN-β therapy for nine days [79]. Data showed that aberrant expression levels of miR-145 and miR-20a-5p were normalized in the whole blood of RRMS patients treated with IFN-β [80]. The pioneer study on the miRNA modulation by GA reported that the administration of GA was able to restore normal levels of miR-146a and miR-142-3p, which were upregulated in PBMCs of the RRMS patients [81]. Later, Singh et al. [82] demonstrated that GA treatment was able to modulate the expression levels of miR-155-5p, miR-27a-3p, miR-9-5p, and miR-350-5p, in plasma and urine exosome, so they were suggested as potential biomarkers of drug response.

The effects of other two DMTs, Natalizumab and Fingolimod, on miRNA profiling have been investigated [83]. Natalizumab, a monoclonal antibody able to prevent immune cell infiltration into the CNS, reduced miR-150 levels in the CSF with a concurrent increase of miR-150 levels in plasma [83]. Fingolimod, an agonist of the sphingosine-1 phosphate receptor (S1P1) that prevents immune cell migration into the brain, showed a divergent pattern in patients who started the treatment, since the plasma levels of miR-150 decreased during the treatment, while the CSF levels remained unchanged [83]. Fingolimod was also reported to restore the blood levels of several miRNAs downregulated in MS patients, including miR-23a, a key regulator of myelination [18], miR-15b, a suppressor of Th17 differentiation, and miR-223, involved in inflammatory processes by targeting STAT5 [84]. Finally, Sáenz-Cuesta et al. [85] demonstrated that the first dose of Fingolimod affects the circulating extracellular vesicles (EVs) in MS patients, where an elevated EV concentration with a dramatic change in their miRNA cargo resulted since the very early administration of the drug.

The expression levels of miR-126 and miR-17 resulted downregulated in CD4+ T cells of RRMS patients under treatment with Natalizumab, while they were upregulated during the clinical relapse, suggesting that the therapeutic effect of Natalizumab may be mediated by these two miRNAs possibly implicated in the pathogenesis of MS [86,87]. In addition, 10 miRNAs resulted differentially expressed in B-lymphocytes obtained from RRMS patients treated with Natalizumab [88]. Two clusters, miR-17∼92 and miR-106b∼25, were particularly deregulated. In another study, 1-year treatment with Natalizumab increased the blood levels of miR-18a, miR-20b, miR-29a, and miR-103 in RRMS patients, all miRNAs downregulated in the CD4+ T-cells of RRMS patients compared to controls [89]. In addition, in the animal model of MS (experimental autoimmune encephalomyelitis, EAE), genetic deletion of the miRNA cluster miR-106a∼363 (containing Natalizumab-regulated miR-20b) resulted in a more severe disease course, and it upregulated in vivo the miR-20b target genes [89]. In addition, the treatment with Natalizumab restored the expression levels of miR-26a and miR-155 [90], which were upregulated in PBMCs of MS patients, as well as in urine exosome, plasma, and in the spinal cord samples from EAE mice [82]. Notably, miR-155 plays a central role in many processes involved in the pathogenesis of MS, such as immune cell activation, neurodegeneration and permeabilization of the BBB [18]. Monocytes from RRMS patients receiving Natalizumab showed reduced expression of the proinflammatory miR-155, compared to untreated MS patients [91]. Similar changes were observed in patients receiving Dimethyl fumarate, which is the first oral first-line drug approved for the treatment of RRMS [91].

Finally, the treatment with Rituximab, a monoclonal antibody that is administered off-label for treating particular MS forms, was able to restore in patients with neuromyelitis optica (NMO) the aberrant levels of brain-specific or brain-enriched miRNAs, including miR-125b, miR-760, miR-135a, miR-134, miR-138, and miR-135b [92].

## 6. MiRNAs Involved in MS Drug Resistance

In the era of Precision Medicine, the big challenge faced by the specialists also in MS is to distinguish between responders and not-responders to a given therapy before starting the treatment and, consequently, to support the clinicians in planning the best therapeutic option available for an individual patient. Treatment of non-responder patients is a critical issue in the clinical management of MS and, unfortunately, it remains a widely unexplored field [18]. Eftekharian et al. [93] investigated for the first time the miRNA involvement in MS drug resistance and showed different miRNA patterns in MS patients responding to Fingolimod compared to non-responders. In their preliminary study, miR-34a-5p and miR-211-5p were found down-regulated in non-responder patients compared to responders, while miR-204-5p was up-regulated [93]. Serum exosomal miRNAs were found to be altered after treatment with Fingolimod [94]. The comparison between stable responders (i.e., patients with no evidence of Gd-enhancing lesions at both baseline and 6-month follow-up) and positive responders (i.e., patients showing active Gd-enhancing lesions at baseline and no enhancing lesions after 6 months of treatment) revealed that the expression levels of two miRNAs (miR-150-5p and miR-548e-3p) decreased with treatment, while the expression of miR-130b-3p, miR-654-5p, and miR-487b-3p increased. Additionally, 11 miRNAs (miR-203a, miR-193a-5p, miR-379-5p, miR-370-3p, miR-382-5p, miR-493-3p, miR-432-5p, miR-485-5p, miR-2110, miR-1307-3p, and miR-1908-5p) were significantly upregulated in stable responders after 6 months of treatment [94]. In line with this study, a recent work profiled the expression of exosome-associated miRNAs in serum of *naïve* and IFN-β-treated groups of MS patients [95]. A total of 16 exosome-associated miRNAs were found differentially expressed in IFN-β-treated RRMS patients with response to therapy compared to those without response. In details, 2 miRNAs (miR-22-3p and miR-660-5p) were upregulated, while 14 (miR-486-5p, miR-451a, let-7b-5p, miR-320b, miR-122-5p, miR-215-5p, miR-320d, miR-19-3p, miR-26a-5p, miR-142-3p, miR-146a-5p, miR-15-3p, miR-23a-3p, and miR-223-3p) were downregulated [95]. Furthermore, the expression level of miR-29b-3p was reported downregulated under IFN-β treatment in RRMS responders versus non-responders, suggesting that the down-regulation of miR-29b-3p may be used as biomarker of discriminating responsiveness [96]. Another recent study has evaluated the expression of the miR-326 in PBMCs of RRMS patients who were responders and non-responders to IFN-β treatment [97]. MiR-26a-5p expression level was found significantly up-regulated in RRMS patients responding to IFN-β, overall suggesting that miRNA profiling may be useful to reduce ineffective treatments [98].

Although to be considered with caution, there are some evidences that miRNAs may be also helpful in the clinical practice as markers of side effects related to the administration of a given DMT in MS. Despite being very effective especially in RRMS patients with high clinical/MRI activity, Natalizumab has been in fact associated with an increased risk of developing progressive multifocal leukoencephalopathy (PML), a severe demyelinating disease of CNS caused by the reactivation of a latent infection of JC virus (JCV) [99]. Muñoz-Culla et al. [99] were the first to explore the blood miRNA signature in order to stratify the individual PML risk. They observed a differential expression of 3 miRNAs (miR-320, miR-320b, and miR-629) between the PML and non-PML groups after 12 months of treatment and suggested that these miRNAs might serve as possible biomarkers for individual PML risk assessment.

## 7. Potential Role of miRNA Polymorphisms in Drug Response and Efficacy

Over the association between miRNomic profiles and their therapeutic effects in NDDs, polymorphisms within or near miRNA binding sites can modify miRNA affinity with the corresponding mRNA targets. This intriguing aspect highlights the existence of “genetic of the epigenetic” contribution to the onset, progression, and inter-individual variations of the drug response [1].

A class of functional polymorphisms, named miR-polymorphisms or miRSNPs, are reported as new player in miRNA-mediated gene regulation [100]. MiRSNPs refer to polymorphisms present at, or near-by, the miRNA binding sites of their target genes as well as in those genes that are involved in miRNA biogenesis [100]. MiRSNPs have been shown to influence the drug response by affecting the expression of drug target genes, mostly in the field of cancer chemotherapy response and survival [101].

A great number of miR-polymorphisms have been associated with many diseases, including CNS-related disorders [102]. As an example, association between miR-146a and polymorphisms of its target gene, interleukin receptor associated kinase 1 (IRAK1), contributed to the susceptibility of MS patients [103]. Notably, Zhang et al. [104] reported that miR-146a promoted remyelination in CNS via IRAK1, and that miRIDIAN miR-146a mimic treatment (by Dharmacon) significantly decreased the level of the protein IRAK1 [104]. The presence of genetic variations in IRAK1, which affect the interaction between miR-146a and its target gene, can potentially lead to different interindividual drug response, in different pathologies. As an example, Wang et al. [105] showed that variations within the miR-433 target site of the gene fibroblast growth factor 20 (FGF20) inhibited the miRNA-target interaction and, as a consequence, increased FGF20 expression level. This genetic variation conferred an higher risk of PD by enhancing the levels of the protein α-synuclein that is the first gene identified as associated with the disease [106].

Ghanbari et al. [107] performed a genome-wide investigation to identify genetic variants in miRNAs and in miRNA-binding sites that are associated with AD. Variants that are located in the seed-matching regions of target genes interfered with the interaction between miRNAs and their target genes, pointing to a function in the drug metabolism and in phenotypic variation [107].

The implication of miRNA variants in gene network and how they can affect the metabolism and efficacy of drugs in NDDs patients remain to be explored.

## 8. The Therapeutic Potential of miRNAs

Recently, a new focus has been added in the field of pharmacoepigenomics: The development of therapeutic epidrugs. In fact, it is now evident that epigenetic status not only can influence the drug response, but it can also be modulated by drugs [108]. Epigenetic therapy, defined as the use of drugs to treat or prevent epigenetic defects associated with a given disease, may represent a step forward the treatment of diseases in which epigenetic regulation plays a role [22,108]. To date, the most studied epidrugs are the DNA methyltransferase inhibitors (DNMTi), the histone acetyltransferase inhibitors (HATi/KATi), and the histone deacetylase inhibitors (HDACi). The Food and Drug Administration (FDA) approved two classes of epidrugs, DNMTi and HDACi, for clinical use in a plethora of diseases, such as cancer, epilepsy, hypertension, and cardiac arrhythmia [108].

In addition to these epidrugs, miRNAs are also gaining attention for their therapeutic potential, and in fact miRNA-based drugs are currently in preclinical phase or in phase 1 and phase 2 clinical trials, mostly in cancer treatment [109]. MiRNAs showed to regulate the expression of efflux and uptake drug transporters and enzymes, with consequent impact on the drug efficacy [21]. Meanwhile, some miRNAs may directly reduce the protein outcome of pharmacological targets and thus control the disease progression. To modulate miRNA expression levels, there are currently two therapeutic strategies: miRNA mimic (agonist) and anti-miRNA (antagomiR) [4,110]. The first one is used to therapeutically restore the concentration of a specific miRNA and, as a result, to down-regulate the expression of specific target/s involved in the disease pathogenesis. Inversely, antagomiR is used to create a loss-of-function in the miRNA of interest [109,111]. Although to date no such miRNA-drugs have been entered into the clinicaltrials.gov database for phase 3 trials, there are active trials (early phase) whose main purpose is to investigate novel miRNA drug candidates. Indeed several Biotech Companies and Pharmaceutical Industries focus exclusively on advancing miRNA-related drug pipelines, such as Miragen Therapeutics, Regulus Therapeutics, ENGeneIC, and Abivax [111]. MiRagen Therapeutics developed MRG-107, a synthetic miRNA antagonist targeting the pro-inflammatory miR-155, whose expression is elevated in the spinal cord of both familial and sporadic ALS patients. In pre-clinical models of ALS, inhibition of miR-155 restored microglial function and prolonged cellular survival.

## 9. Conclusions

The emerging advances in the field of miRNA pharmacoepigenomics open up new possibilities for the development of a precision medicine approach for many untreatable progressive NDDs. To date, this apparently futuristic approach has solid foundation in several pharmacological studies. In fact, it has been demonstrated that some FDA-approved drugs are able to restore selected miRNAs that resulted altered in NDDs, as well as miRNAs can regulate the expression of pharmacology-related target genes through direct interaction.

The knowledge of miRNA pharmacoepigenomics not only add knowledge about the interindividual variability in drug disposition and response, but also offers new clues to develop more effective treatments.

Nevertheless, the use of miRNAs as potential therapeutic targets remains controversial and still need to be investigated especially for the methods of delivery and the target specificity, especially in case of NDDs.

## Figures and Tables

**Table 1:** List of miRNAs involved into therapeutic effects of available conventional (and non-conventional) treatments for some NDDs. For each disease, the investigated drug, the differentially expressed miRNAs, their source, the type of comparison and the published papers are indicated.

Disease	Drug	miRNA	Source	Comparison	Ref.
Alzheimer’s Disease	Donepezil	miR-206-3p	Hippocampus, cortex - Mouse	Treat vs Non treat	[43]
	Simvastatin	miR-106b	SH-SY5Y cells; APP/PS1 mice brain tissues	Treat vs Non treat	[47]
	Osthole	miR-9	APP-overexpressed cells	Treat vs Non treat	[45,54]
		miR-107	APP-overexpressed cells; APP/PS1 mice	Treat vs Non treat	[48]
		miR-101a-3p	APP-overexpressed cells; APP/PS1 mice	Treat vs Non treat	[52]
	AGR-GRg1	miR-873-5p	Hippocampus - Mouse	Treat vs Non treat	[55]
Parkinson’s disease	l-Dopa	miR-30b-5p, miR-30a-5p	Plasma	Treat vs Non treat	[69]
		miR-29a-3p, miR-30b-5p, miR-103a-3p	PBMCs	Treat vs Non treat	[62]
		miR-16-2-3p, miR-26a-2-3p, miR-30a	Peripheral blood	Treat vs Non treat	[58]
		miR-155	PBMCs	Treat vs Non treat	[61]
	l-Dopa Dopamine receptor agonists Amantadine	miR-7, miR-9-3p, miR-9-5p, miR-129, miR-132	Peripheral blood	Treat vs Non treat	[70]
Multiple Sclerosis	Interferon-β	miR-29	PBMCs	Treat vs Non treat	[78]
		miR-145, miR-20a-5p	Whole blood	Treat vs Non treat	[80]
		miR-22-3p, miR-660-5p, miR-486-5p, miR-451a, let-7b-5p, miR-320b, miR-122-5p, miR-215-5p, miR-320d, miR-19-3p, miR-26a-5p, miR-142-3p, miR-146a-5p, miR-15-3p, miR-23a-3p, miR-223-3p	Exosome	Res vs Non res	[95]
		miR-29b-3p	PBMCs	Res vs Non res	[96]
		miR-326	PBMCs	Res vs Non res	[97]
		miR-26a-5p	PBMCs	Res vs Non res	[98]
	Glatiramer acetate	miR-146a, miR-142-3p	PBMCs	Treat vs Non treat	[81]
		miR-155-5p, miR-27a-3p, miR-9-5p, miR-350-5p	Plasma and urine exosome	Treat vs Non treat	[82]
	Natalizumab	miR-150	CSF, Plasma	Treat vs Non treat	[83]
		miR-126, miR-17	CD4 + T cells	Treat vs Non treat	[86,87]
		miR-17~92, miR-106b~25	B lymphocytes	Treat vs Non treat	[88]
		miR-18a, miR-20b, miR-29a, miR-103	Blood, CD4 + T cells	Treat vs Non treat	[89]
		miR-26a, miR-155	PBMCs	Treat vs Non treat	[90]
		miR-155	Monocytes	Treat vs Non treat	[91]
	Dimethyl fumarate	miR-155	Monocytes	Treat vs Non treat	[91]
	Fingolimod	miR-150	Plasma	Treat vs Non treat	[83]
		miR-23a, miR-15b, miR-223	Blood	Treat vs Non treat	[84]
		miR-34a-5p, miR-211-5p, miR-204-5p	Peripheral blood	Res vs Non res	[93]
		miR-150-5p, miR-548e-3p, miR-130b-3p, miR-654-5p, miR-487b-3p, miR-203a, miR-193a-5p, miR-379-5p, miR-370-3p, miR-382-5p, miR-493-3p, miR-432-5p, miR-485-5p, miR-2110, miR-1307-3p, miR-1908-5p	Serum exosomal	Stable res vs Positive res	[94]
Progressive Multifocal Leukoencephalopathy	Natalizumab	miR-320, miR-320b, miR-629	Blood	Treat vs Non treat	[99]
Neuromyelitis Optica	Rituximab	miR-125b, miR-760, miR-135a, miR-134, miR-138, miR-135b	Blood	Treat vs Non treat	[92]
